# Internally displaced persons’ ed utilization and disease patterns: A single center retrospective chart review

**DOI:** 10.1371/journal.pone.0333245

**Published:** 2025-12-29

**Authors:** Eveline Hitti, Ghada Chamandi, Hiba Fadlallah, Hani Tamim, Maha Makki, Afif Mufarrij

**Affiliations:** 1 Department of Emergency Medicine, American University of Beirut Medical Center, Beirut, Lebanon; 2 Clinical Research Institute, Faculty of Medicine, American University of Beirut Medical Center, Beirut, Lebanon; 3 Department of Internal Medicine, American University of Beirut Medical Center, Beirut, Lebanon; Universitair Kinderziekenhuis Koningin Fabiola: Hopital Universitaire des Enfants Reine Fabiola, BELGIUM

## Abstract

**Background:**

Armed conflicts profoundly disrupt healthcare systems, with internally displaced persons (IDPs) facing distinct challenges and barriers to accessing healthcare. This study explores emergency department (ED) utilization patterns, disease spectrum, and patient outcomes of IDPs during conflict in comparison with non-IDPs (NIDPs).

**Methods:**

A retrospective chart review was conducted on all patients presenting to the American University of Beirut Medical Center between October 7, 2024, and November 27, 2024. Patients were categorized into the IDPs and NIDPs groups based on documented residential status at the time of presentation. Specifically, we compared the demographic data, ED visit characteristics, and disease spectrums in displaced versus non-displaced patients in a tertiary care hospital in Lebanon.

**Results:**

A total of 5686 patients were reviewed, of which 776 and 4910 patients were identified as IDPs and NIDPS, respectively. Displaced patients tended to be younger (p < 0.001), insured (p < 0.001), and were more likely to arrive by ambulance (p < 0.001). A greater proportion of IDPs left without being seen, against medical advice, or were transferred to another hospital. Moreover, a higher proportion of IDPs who were admitted required critical care (CC) admission. Displacement, age, and guarantor type (3^rd^ party coverage) emerged as distinct variables associated with the need for CC admission (p < 0.001, p = 0.02, p < 0.001, respectively).

**Conclusion:**

ED utilization patterns of IDPs have distinct features that reflect the challenges they face regarding healthcare access as well as the higher complexity of cases, increased CC needs, and distinct disease spectrums for ED visits. These results highlight the importance of tailored health interventions for IDPs during times of conflict that address these challenges.

## Introduction

Armed conflicts profoundly disrupt healthcare systems, with a vast literature documenting their adverse effects on access to healthcare, with displaced populations facing the greatest barriers to care [1]. While many individuals cross international borders and seek refuge, millions of individuals globally are forced to flee their homes but remain within their country’s borders, resulting in a large number of internally displaced persons (IDPs) [2]. The majority of IDPs reside in low- and middle-income countries (LMICs) [3] and face distinct health vulnerabilities, including inadequate access to healthcare, deteriorating living conditions, as well as increased exposure to communicable diseases [4,5]. Studies that have examined IDPs’ health outcomes suggest that they experience worse health outcomes than both refugee and host populations [6–10]. Despite these documented vulnerabilities, IDPs remain critically understudied in conflict health research, with most literature focusing either solely on refugees or on the entire population.

The Emergency Department (ED) commonly serves as a vital healthcare safety net in conflict-affected areas, taking on not only trauma patients but also patients with chronic disorders and preventable acute illnesses [11]. While prior studies have explored ED utilization patterns of refugees and migrant and non-migrant populations during wars [3], most of these studies have aggregated refugees, IDPs, and non-IDPs, without exploring these patterns in IDPs specifically or the differences between IDPs and NIDPs [3]. Accordingly, inequalities in health needs and care-seeking behaviors could potentially be obscured [12]. To our knowledge, no studies have systematically compared ED utilization patterns or illness spectrums between IDPs and NIDPs during active conflict.

Previous studies have explored refugees’ ED utilization patterns, with most of these studies reporting that refugees face unique challenges with access to healthcare, present with a higher proportion of low acuity/nonurgent visits, and have lower overall rates of hospital admission [9,10]. While there are no studies exploring ED utilization patterns in IDPs, few studies have explored health outcomes, health needs, and quality of life of IDPs in general during times of conflict. These studies reported a higher incidence of communicable diseases, particularly among displaced populations living in shelters, as well as a higher incidence of non-communicable diseases. Nevertheless, studies exploring ED utilization patterns of IDPs are lacking [13].

Lebanon, a low-resource setting, provides a relevant and urgent context for studying this issue. In 2024, the country experienced a series of violent events, including armed clashes, bombings, and

political unrest, resulting in widespread destruction, civilian casualties, and a significant increase in internal displacement, particularly in urban centers like Beirut and its outskirts. The largest wave of mass displacement occurred in the Fall of 2024 when Israel initiated the “Northern Arrows” operation with escalated attacks on Lebanon, leading to an estimated 358,198 displaced individuals escaping the heavily targeted areas, placing extraordinary pressure on already overstretched services [14–16].

This study aims to assess the impact of the recent armed conflict on IDPs visiting the ED and compare their presentations to those of NIDPs during the same period to explore the differentiated impact of the conflict on this population in a single-center hospital in Beirut, Lebanon.

## Materials and methods

### Study design

A secondary analysis of ED administrative data was performed to compare emergency department (ED) utilization patterns, disease spectrum, and patient outcomes of IDPs during conflict in comparison with non-IDPs (NIDP) between October 7, 2024, and November 27, 2024.

### Study setting

This study was conducted at the American University of Beirut Medical Center (AUBMC) ED, a 384-bed tertiary care teaching hospital situated in Beirut, Lebanon. The ED ranks among the largest emergency departments in the country, managing approximately 57,000 visits each year. Given its central location, the hospital serves as a prominent urban teaching facility and referral center. The core team includes Emergency Medicine specialists, as well as experienced physicians who may not have specific emergency training but possess extensive expertise in the field. The ED is divided into high acuity, low acuity, and pediatric areas, thereby allowing for optimal provision of care to a diverse variety of patients. Patients are triaged into the various sections based on a combination of the Emergency Severity Index (ESI) algorithm as well as specific clinical criteria. On October 7, 2024 – two weeks after the start of Operations Arrows – the ED introduced a screening question to identify IDPs at triage that was captured by nurses and integrated into the electronic medical record system.

The ED at AUBMC follows a hybrid point of service collection model: Patients triaged as low acuity (ESI 4 or 5) are asked to cover an upfront facility and professional fee charge after medical screening. Higher acuity patients, however (ESI 1, 2, or 3), are evaluated fully and stabilized prior to financial clearance. Moreover, charity care funds and financial counseling are available for higher acuity cases where patients are unable to cover the full costs of care [16]. During the study period, the Lebanese Ministry of Public Health designated public hospitals as the primary referral centers for IDPs. A list of these centers was offered to all IDPs who chose to leave without being seen at the AUBMC-ED. For higher acuity patients (ESI 1,2, or 3) who, after initial management and stabilization at our ED, were recommended admission but were unable to afford inpatient care, transfer center services were offered to assist with safely transferring patients to the designated public hospitals.

This study received approval from the American University Institutional Review Board under protocol number (BIO-2024–0514). Requirement for informed consent was waived by the ethics committee at our institution due to the retrospective nature of the study.

### Participants

All adult and pediatric patients who visited the ED during the previously specified period were included in the analysis and subsequently classified as IDPs and NIDPs.

### Data collection and measurement

De-identified data were retrieved from the AUBMC-ED administrative database on 15/08/2025. All data were anonymized prior to analysis, and the authors did not have access to information that could identify individual participants during or after data collection. Data included variables such as patient demographics (age, gender, nationality, and marital status) as well as ED visit characteristics, including the Emergency Severity Index (ESI), length of stay (LOS), ED disposition, guarantor information, and ICD-10 diagnosis codes. The discharge diagnoses, based on the International Classification of Diseases, Tenth Revision (ICD-10), were systematically aggregated into 23 Major Diagnostic Categories to facilitate data analysis and interpretation.

### Statistical analysis

Data entry, management, and analysis were conducted using the Statistical Package for Social Sciences (SPSS) version 24.0. Descriptive statistics for categorical variables were expressed as numbers and percentages, while means and standard deviations (SD) were used for continuous variables. Comparisons between displaced and non-displaced populations, as well as those for CC admissions, were evaluated using the Pearson chi-square test for categorical variables and the Student’s t-test for continuous variables. Additionally, **ENTER** multivariate logistic regression was used to assess the relationship between CC needs, displacement status, and the different predictors. Factors that we found clinically and statistically significant were included in the model. Moreover, factors shown to have multicollinearity, as tested by variance inflation factor (VIF), were reduced to one. Specifically, the ESI and LOS were found to be highly corelated; thus, the LOS was considered in the model. Results were reported as Odds Ratios (OR) with 95% confidence intervals (CI), and a p-value of < 0.05 was considered statistically significant.

## Results

Table 1 represents the demographic data and ED visit characteristics of patients based on displacement status. A total of 776 IDPs and 4,910 non-IDPs were included in the final analysis.

**Table 1:** Comparison of Patient Demographics and ED Visit Characteristics Between the displaced and non-displaced population during the specified period.

		Displaced		
		No N = 4910	Yes N = 776	p-value
**Patient Demographics**				
**Age**	Mean (±SD)	41.44 ± 25.65	34.62 ± 26.15	<0.001
**Age Categories**	1-17 years	941 (19.2%)	248 (32.0%)	<0.001
	18-44 years	1844 (37.6%)	242 (31.2%)	
	45-64 years	988 (20.1%)	154 (19.8%)	
	65 years and older	1137 (23.2%)	132 (17.0%)	
**Gender**	Male	2598 (52.9%)	389 (50.1%)	0.15
	Female	2311 (47.1%)	387 (49.9%)	
**Nationality**	Non-Lebanese	295 (6.1%)	23 (3.2%)	0.002
	Lebanese	4572 (93.9%)	698 (96.8%)	
**Marital status**	Single	2554 (52.4%)	495 (67.3%)	<0.001
	Married	2185 (44.9%)	226 (30.7%)	
	Other (widowed, divorced)	131 (2.7%)	14 (1.9%)	
**Guarantor**	Self-Payer	907 (19.1%)	0 (0.0%)	<0.001
	Insurance or 3rd party payment	3839 (80.9%)	685 (100.0%)	
**ED Visit Characteristics**				
**Mode of arrival**	Self-transport	3005 (90.6%)	493 (81.9%)	<0.001
	Ambulance	313 (9.4%)	109 (18.1%)	
**Emergency Severity Index (ESI)**	High	407 (8.4%)	120 (15.5%)	<0.001
	Intermediate	4056 (83.3%)	597 (76.9%)	
	Low	406 (8.3%)	59 (7.6%)	
**LOS, hours**	Mean (±SD)	3.27 ± 4.67	3.85 ± 7.22	0.03
**ED Disposition**	Home	3352 (69.1%)	495 (65.0%)	<0.001
	AMA	220 (4.5%)	115 (15.1%)	
	Admit	1086 (22.4%)	49 (6.4%)	
	**General**	884 (81.4%)	30 (61.2%)	
	**Critical care**	202 (18.6%)	19 (38.8%)	
	Transfer to another facility	13 (0.3%)	11 (1.4%)	
	**General**	12 (92.3%)	9 (81.8%)	
	**Critical care**	1 (7.7%)	2 (18.2%)	
	Dead	23 (0.5%)	3 (0.4%)	
	LWBS	156 (3.2%)	89 (11.7%)	
**Labs done**	Yes	3003 (61.2%)	441 (56.8%)	0.02
**Electrocardiogram**	Yes	1721 (35.1%)	260 (33.5%)	0.40

*SD: Standard deviation, ESI: Emergency severity index, LOS: Length of stay, ED: Emergency department, AMA: against medical advice, LWBS: Left without being seen.

The mean age of IDPs was significantly lower than that of non-IDPs (34.62 ± 26.15 vs 41.44 ± 25.65 years, p < 0.001). There was no significant difference in gender distribution between the two groups (p = 0.15). While most patients in both groups were of Lebanese nationality, the proportion of Lebanese patients was greater in the IDP group compared to the non-IDP group (96.8% vs 93.9%, p = 0.002). Moreover, a greater proportion of IDPs had insurance or 3^rd^ party payments (100.0% in the IDP group vs 80.9% in the non-IDP group, p-value <0.001). Although the primary mode of arrival for both groups was by self-transport, a greater proportion of patients in the IDP group arrived by ambulance as compared to the non-IDP group (81.9% vs 90.6%, p < 0.001). The emergency severity index (ESI) distribution also varied significantly between groups. A greater proportion of displaced patients were of higher acuity (ESI 1 and 2) compared to non-displaced patients (15.5% vs. 8.4%, p < 0.001). Moreover, the length of stay was also significantly higher among IDPs (3.85 h ± 7.22) compared to non-IDPs (3.27h ± 4.67 hours) (p = 0.03). Notably, a greater proportion of IDPs left without being seen (LWBS), against medical advice (AMA), or were transferred to another facility. Additionally, although a lower proportion of IDPs were admitted, of those who required admission, IDPs were significantly more likely to require critical care admissions than non-IDPs (38.8% vs 18.6%, p < 0.001). Similarly, of those who were transferred to another facility, IDPs were still significantly more likely to require critical care admissions in the outside hospital (18.2%vs 7.7%, p < 0.001).

Table 2 represents a comparison of patient demographics and ED visit characteristics for patients who were admitted to critical care (CC) compared to those who were admitted under general inpatient care (GC). During this period, 250 patients either passed away or were admitted to critical care (CC group), while 935 were admitted under general care (GC group).

**Table 2:** Comparison of patient demographics and ED visit characteristics for patients admitted to critical care.

		Critical care		
		No N = 935	Yes N = 250	p-value
**Patient Demographics**				
**Age**	Mean (±SD)	56.25 ± 26.97	60.64 ± 25.85	0.009
**Age Categories**	1-17 years	113 (12.1%)	25 (10.0%)	
	18-44 years	187 (20.0%)	29 (11.6%)	
	45-64 years	181 (19.4%)	54 (21.6%)	
	65 years and older	454 (48.6%)	142 (56.8%)	
**Gender**	Male	494 (52.8%)	141 (56.6%)	0.29
	Female	441 (47.2%)	108 (43.4%)	
**Nationality**	Non-Lebanese	58 (6.2%)	22 (8.8%)	0.15
	Lebanese	877 (93.8%)	228 (91.2%)	
**Marital status**	Single	291 (31.1%)	75 (30.0%)	0.22
	Married	590 (63.1%)	167 (66.8%)	
	Other (widowed, divorced)	54 (5.8%)	8 (3.2%)	
**Guarantor**	Self-Payer	146 (15.6%)	71 (28.4%)	<0.001
	Insurance or 3rd party payment	787 (84.4%)	179 (71.6%)	
**ED Visit Characteristics**				
**Displaced**	Yes	39 (4.2%)	24 (9.6%)	<0.001
**Mean of arrival**	Self-transport	525 (77.7%)	121 (66.5%)	0.002
	Ambulance	151 (22.3%)	61 (33.5%)	
**Emergency Severity Index (ESI)**	High	151 (16.1%)	149 (59.8%)	<0.001
	Intermediate	781 (83.5%)	100 (40.2%)	
	Low	3 (0.3%)	0 (0.0%)	
**LOS, hours**	Mean (±SD)	5.80 ± 4.67	7.18 ± 11.11	0.03
**Labs done**	Yes	906 (96.9%)	226 (90.4%)	<0.001
**Electrocardiogram**	Yes	553 (59.1%)	197 (78.8%)	<0.001

*SD: Standard deviation, ED: Emergency department, ESI: Emergency severity index, LOS: Length of stay.

There was no significant difference in gender distribution between the two groups (p = 0.29). Moreover, a higher proportion of patients were of Lebanese nationality in both groups. However, this proportion was greater in the IDP group (91.2% vs 93.8%, p = 0.15).

A significantly higher portion of patients in the CC group were self-payers compared to those in the GC group (28.4% vs 15.6%, p < 0.001).

The major mode of arrival for the CC group was by self-transport (66.5% vs 77.7%, p < 0.001). The ESI distribution varied significantly, with a greater proportion of patients who required CC admission being of higher acuity upon presentation (ESI 1 & 2) (59.8% vs. 16.1%, p < 0.001). Moreover, the LOS was significantly higher in the CC group compared to the GC group (7.18 h ± 11.11 vs. 5.80h ± 4.67 hours p < 0.001).

The ENTER multivariate logistic regression analysis (Table 3) included all clinically relevant and statistically significant variables associated with the need for critical care, namely, displacement status, gender, age, guarantor, LOS, nationality, and marital status. According to the analysis, displacement status was a strong and statistically significant predictor of the need for critical care admission (OR 2.90, 95% CI: 1.66–5.05 p < 0.001). Older age was also associated with higher odds of requiring critical care admission (OR of 1.09, 95% CI: 1.02–1.17, p = 0.02). Moreover, having insurance or 3^rd^ party payment was significantly associated with lower odds of critical care admission (OR: 0.42, 95% CI: 0.30–0.60, p < 0.001).

**Table 3:** ENTER multivariate logistic regression of predictors of critical care.

	Critical care (reference: no)	
	**OR (95% CI)**	**P-value**
**Displaced – Yes**	2.90 (1.66–5.05)	<0.001
**Age, years**	1.09 (1.02–1.17)	0.02
**LOS, hours**	1.15 (0.95–1.38)	0.15
**Gender,** Female	0.87 (0.65–1.16)	0.34
**Nationality,** Lebanese	0.80 (0.47–1.39	0.43
**Marital status,** Married	1.03 (0.70–1.52)	0.88
**Guarantor,** Insurance or 3^rd^ party payment	0.42 (0.30–0.60)	<0.001
Variables included in the model were: Displaced (reference: no); Gender (reference: male); Age (increase by 10 years), Guarantor (reference: self-payer); LOS (increase by 10 hours); Nationality (reference: non-Lebanese); Marital status (reference: Single)		

*OR: Odds Ratio, CI: Confidence interval, ESI: Emergency severity index

Table 4 illustrates the different diagnoses that patients in the study were found to have by the end of the ED visit. Patients who LWBS were excluded from this table since they were not seen by a physician and therefore their diagnosis could not be determined. The IDP group was more likely to have diseases of the blood and blood-forming organs (OR 2.11, 95% CI: 1.13–3.95) and mental, behavioral, and neurodevelopmental disorders (OR: 2.57, 95% CI: 1.68–3.93). They were less likely to seek healthcare services due to factors influencing health status (OR: 0.36, 95% CI: 0.18–0.74). Additionally, a lower proportion of displaced patients experienced injury, poisoning, and certain other consequences from external causes (OR: 0.84, 95% CI: 0.68–1.05).

**Table 4:** Comparison of ED diagnosis among displaced and non-displaced patients.

	Displaced			
CCS	No N = 4754	Yes N = 687	OR (95%CI)	P-Value
Diseases of the blood and blood-forming organs, and certain disorders involving the immune mechanism	43 (0.9%)	13 (1.9%)	2.11 (1.13–3.95)	0.02
Diseases of the circulatory system	375 (7.9%)	61 (8.9%)	1.14 (0.86–1.51)	0.37
Diseases of the digestive system	542 (11.4%)	89 (12.9%)	1.16 (0.91–1.47)	0.25
Diseases of the ear and mastoid process	75 (1.6%)	6 (0.9%)	0.55 (0.24–1.28)	0.18
Endocrine, nutritional, and metabolic diseases	42 (0.9%)	3 (0.4%)	0.50 (0.15–1.60)	0.36
External causes of morbidity	84 (1.8%)	17 (2.5%)	1.41 (0.83–2.39)	0.22
Diseases of the eye and adnexa	37 (0.8%)	6 (0.9%)	1.12 (0.47–2.67)	0.82
Factors influencing health status and contact with health services	150 (3.2%)	8 (1.2%)	0.36 (0.18–0.74)	0.002
Diseases of the genitourinary system	352 (7.4%)	48 (7.0%)	0.94 (0.69–1.28)	0.70
Certain infectious and parasitic diseases	174 (3.7%)	22 (3.2%)	0.87 (0.55–1.37)	0.55
Injury, poisoning, and certain other consequences of external causes	876 (18.4%)	110 (16.0%)	0.84 (0.68–1.05)	0.13
Congenital malformations, deformations, and chromosomal abnormalities	9 (0.2%)	0 (0.0%)	0.87 (0.86–0.88)	0.61
Mental, behavioral, and neurodevelopmental disorders	83 (1.7%)	30 (4.4%)	2.57 (1.68–3.93)	<0.001
Diseases of the musculoskeletal system and connective tissue	292 (6.1%)	14 (2.0%)	0.32 (0.18–0.55)	<0.001
Neoplasms	57 (1.2%)	3 (0.4%)	0.36 (0.11–1.16)	0.07
Diseases of the nervous system	144 (3.0%)	16 (2.3%)	0.76 (0.45–1.29)	0.40
Certain conditions originating in the perinatal period	8 (0.2%)	3 (0.4%)	2.60 (0.69–9.83)	0.15
Pregnancy, childbirth, and the puerperium	20 (0.4%)	6 (0.8%)	2.08 (0.84–5.21)	0.11
Diseases of the respiratory system	360 (7.6%)	58 (8.4%)	1.12 (0.84–1.50)	0.42
Diseases of the skin and subcutaneous tissue	105 (2.2%)	11 (1.6%)	0.72 (0.39–1.35)	0.30
Symptoms, signs, and abnormal clinical and laboratory findings, not elsewhere classified	895 (18.8%)	158 (23.0%)	1.29 (1.06–1.56)	0.01

*Patients who LWBS were excluded from this analysis

*CCS: Clinical Classification software, OR: Odds ratio, CI: Confidence interval.

The displaced group also had a lower incidence of diseases of the musculoskeletal system and connective tissue compared to the non-displaced group (OR: 0.32, 95% CI: 0.18–0.55). Moreover, the odds of having certain conditions originating in the perinatal period (OR: 2.60, 95% CI: 0.69–9.83), as well as during pregnancy, childbirth, and the puerperium (OR: 2.08, 95% CI: 0.84–5.21) were higher in IDPs than NIPDs. However, these findings were not statistically significant.

## Discussion

In this study, we investigated the ED utilization patterns, disease spectrums, and patient outcomes of IDPs compared to NIDPs at a single center during a major conflict that led to mass internal displacement in Lebanon. Displaced patients tended to be younger, insured or have 3^rd^ party coverage, and were more likely to arrive by ambulance compared to non-displaced patients. Moreover, a greater proportion of IDPs LWBS, AMA, or were transferred to another hospital.

This study is unique in its focus on the demographics and ED utilization patterns of IDPs [5,16]. By identifying IDPs upon presentation through the implementation of a screening method at triage, we were able to acquire data on IDPs. This unique approach enabled us to investigate utilization patterns among IDPs in a manner not previously explored in prior research. Since this screening method is not a standard component of ED tools, other studies have typically depended on population-based surveys or visits to displaced shelters, none of which allowed for the examination of ED utilization patterns in this specific population [5]. Moreover, most studies have focused on refugees for similar reasons, as they faced comparable challenges in capturing the displacement status of patients presenting to EDs [17,18]. Our distinct advantage was the rapid integration of a screening tool in the ED shortly after displacement occurred. This allowed nurses to capture the data regarding displacement during triage, after which the data was incorporated into our information system and subsequently analyzed.

Our study found that IDPs tended to be younger and were more likely to be insured or have 3^rd^ party payments/coverage, which was consistent with previous research on displaced populations. In this study, 3^rd^ party coverage entails coverage by the institution, since most of these patients could not cover the cost of care. A higher portion of IDP patients LWBS, AMA, or were transferred to another facility. Within the context of a private hospital, we believe that the main driver for patients who LWBS or AMA was the financial barriers to care. This is consistent with previous studies, which found that non-compensable coverage status was the strongest predictor for LWBS in patients presenting to the ED [18]. Moreover, in a previous study, non-compensable status was the strongest predictor of LWBS or AMA [18].

Displacement was strongly significantly associated with a greater need for CC admissions. Moreover, IDPs had a longer LOS than non-IDPs, although this difference was not significant. The higher proportion of IDPs arriving by ambulance further supports the notion of increased acuity or urgent needs. In fact, displacement emerged as an independent variable associated with the need for critical care admission. This is a critical finding, indicating that IDPs are presenting to the ED with more severe conditions, potentially due to limited or delayed access to preventative services or disruptions in care related to displacement. This is an area that needs further exploration to develop strategies to mitigate the impact of displacement on the health outcomes of patients.

Regarding disease spectrums, IDPs were more likely to present with mental health and behavioral disorders, while NIDPs showed a higher incidence of injuries, poisoning, and other external causes. The increased prevalence of mental health issues among IDPs is possibly a consequence of displacement and conflict-related trauma [16], highlighting the urgent need for accessible mental health services within displaced communities. The lower rates of injuries and external causes among IDPs could be attributed to the majority of these diagnoses falling in the low acuity triage category, which, within our system, requires financial clearance after triage, with many patients selecting to leave to the designated public service referral centers, without completing service at our ED. While not statistically significant due to low overall case numbers, the observed higher odds of perinatal and pregnancy-related conditions among IDPs also warrant further investigation, as these are vulnerable populations with specific healthcare needs that may be unmet during displacement [10,16]. While no other studies have explored the disease spectrum of IDPs’ ED visits during conflict specifically, our study affirms the findings of other studies that report increases in overall mental health issues, communicable diseases, and non-communicable diseases in refugee populations during war [6,9,10].

Our study’s findings align with the broader understanding of health disparities in conflict-affected populations [12,19,20]. Previous studies exploring disease patterns in IDPs and refugees reported similar trends in non-communicable diseases in displaced populations, as well as an increase in severity of these conditions at the time of presentation, likely due to their displaced status preventing them from seeking medical attention at an earlier time [4]. However, unlike in our study, several previous studies focused on the increased prevalence of communicable diseases in IDPs and refugees, particularly those living in camps [7]. Studies have found that infectious diseases such as tuberculosis (TB), HIV/AIDS, malaria, cholera, and respiratory infections were significantly more prevalent among IDP populations [7,8]. These studies highlighted that these populations’ living conditions (overcrowded camps and contaminated water supplies) were a key factor causing the rise in communicable disease prevalence [8]. The increased critical care needs among IDPs underscore the profound impact of displacement on health outcomes and the strain it places on emergency healthcare systems. The challenge of capturing data on this population has historically left a major gap in understanding their health care needs [5,10]. The study’s retrospective design, utilizing administrative data from a well-established ED with a structured triage system (ESI algorithm) and clear patient categorization (high acuity, low acuity, pediatric areas), provides a robust foundation for these observations that offers some initial understanding of this vulnerable population’s emergent health care needs.

Future research should delve deeper into the underlying factors contributing to these disparities, such as specific barriers to primary care, access to medications, and a deeper understanding of IDPs’ mental health needs [9,10]. Developing targeted interventions, including community-based healthcare initiatives, mobile clinics, and integrated mental health services, is crucial to address the complex healthcare needs of IDPs and mitigate the adverse effects of conflict on their health. Further prospective studies with longer follow-up periods and a broader geographical scope would provide a more comprehensive understanding of these issues.

This study has several limitations. As a retrospective chart review conducted at a single tertiary care center, the generalizability of our findings to other settings or populations may be limited. The study period, while reflective of a conflict time, was relatively short (October to November 2024). Furthermore, reliance on administrative data means that data on potential confounders and additional clinical details that could influence outcomes may not have been captured. Moreover, the high number of patients who LWBS may have biased the findings since the outcomes of these patients are unknown. Furthermore, LWBS patients do not have a documented end-of-visit diagnosis in the record, and thus were excluded from the part of the analysis comparing ED visit diagnoses. Finally, while financial barriers are a possible explanation for some disparities, direct data on socioeconomic status or specific financial constraints of individual patients were not presented in the provided materials.

## Conclusion

Wartime internal displacement impacts ED utilization patterns of IDPs and patient outcomes, with prolonged ED stays and a greater need for CC admissions among displaced populations. These results emphasize the unique healthcare challenges faced by IDPs and highlight the importance of tailored emergency medicine services and support systems during times of conflict. Future research should focus on exploring the root causes of these healthcare disparities as well as the long-term health impacts of displacement on IDPs. Implementing targeted interventions, such as community-based healthcare initiatives, mobile and decentralized primary healthcare units, and integrated mental health and psychosocial support within general healthcare, is crucial for addressing this population’s unique needs. A more comprehensive understanding requires prospective studies with longer follow-up periods and a broader scope.
