# Correction: Internally displaced persons’ ed utilization and disease patterns: A single center retrospective chart review

**DOI:** 10.1371/journal.pone.0348082

**Published:** 2026-04-27

**Authors:** Eveline Hitti, Ghada Chamandi, Hiba Fadlallah, Hani Tamim, Maha Makki, Afif Mufarrij

In the Results subsection of the Abstract, there is an error in the second sentence. The correct sentence is: Displaced patients tended to be younger (p < 0.001), were less likely to be insured (p < 0.001), and were more likely to arrive by ambulance (p < 0.001).

In the Results subsection of the Abstract, there is an error in the fifth sentence. The correct sentence is: Displacement, age, and length of stay emerged as distinct variables associated with the need for CC admission (p = 0.003, p = 0.03, p = 0.03, respectively).

In the Study setting subsection of the Materials and methods, there is an error in the sixth sentence of the second paragraph. The correct sentence is: For higher acuity patients (ESI 1,2, or 3), initial ED management and stabilization was covered through charity care funds (Displacement Fund) earmarked for emergent care of displaced patients. After ED stabilization, patients who required admission but were unable to afford inpatient care were supported through the hospital’s transfer center services to secure safe transfer to the designated public hospitals.

In the Statistical Analysis subsection of the Materials and methods, there is an error in the seventh sentence. The correct sentence is: Specifically, the ESI and LOS were found to be highly corelated; thus, the LOS was considered in the model. Results were reported as Odds Ratios (OR) with 95% confidence intervals (CI), and a p-value of < 0.05 was considered statistically significant.

Tables 1 to 3 were uploaded incorrectly. Please see the correct Tables 1–3 here.

In the Results, there is an error in the fourth sentence of the second paragraph. The correct sentence is: Moreover, a greater proportion of non-IDPs were insured compared to IDPs (79.6% in the non-IDP group vs 6.0% in the IDP group, p-value <0.001).

In the Results, there is an error in the fifth paragraph. The correct paragraph is: A significantly higher portion of patients in the CC group were covered by the displacement fund (4.8% vs. 1.6%, p < 0.001) or were self-payers (32.4% vs. 20.5%, p < 0.001) compared to those in the GC group. Conversely, a greater proportion of patients in the GC group were insured compared to those in the CC group (77.9% vs. 62.8%, p < 0.001).

In the Results, there is an error in the seventh paragraph. The correct paragraph is: The ENTER multivariate logistic regression analysis (Table 3) included all clinically relevant and statistically significant variables associated with the need for critical care, namely, displacement status, gender, age, guarantor, LOS, nationality, displacement status, age, LOS, gender, nationality, and marital status. According to the analysis, displacement status was a strong and statistically significant predictor of the need for critical care admission (OR 2.27, 95% CI: 1.32–3.92, p = 0.003). Older age was also associated with higher odds of requiring critical care admission (OR 1.08, 95% CI: 1.01–1.16, p = 0.03). Moreover, longer LOS was also a significant predictor of the need for CC admission (OR 1.24 95% CI 1.02–1.50, p = 0.03).

In the Discussion, there is an error in the second sentence of the first paragraph. The correct sentence is: Displaced patients tended to be younger, were less likely to be insured, and were more likely to arrive by ambulance compared to non-displaced patients*.*

In the Discussion, there is an error in the first sentence of the third paragraph. The correct sentence is: Our study found that IDPs tended to be younger and were less likely to be insured. These findings were consistent with previous research on displaced populations.

In the Discussion, there is an error in the second sentence of the fourth paragraph. The correct sentence is: Moreover, IDPs had a longer LOS than non-IDPs, although this difference was not significant.

**Table 1:** Comparison of Patient Demographics and ED Visit Characteristics Between the displaced and non-displaced population during the specified period.

		Displaced		
		No N = 4910	Yes N = 776	p-value
**Patient Demographics**				
**Age**	Mean (±SD)	41.44 ± 25.65	34.62 ± 26.15	<0.001
**Age Categories**	1-17 years	941 (19.2%)	248 (32.0%)	<0.001
	18-44 years	1844 (37.6%)	242 (31.2%)	
	45-64 years	988 (20.1%)	154 (19.8%)	
	65 years and older	1137 (23.2%)	132 (17.0%)	
**Gender**	Male	2598 (52.9%)	389 (50.1%)	0.15
	Female	2311 (47.1%)	387 (49.9%)	
**Nationality**	Non-Lebanese	295 (6.1%)	23 (3.2%)	0.002
	Lebanese	4572 (93.9%)	698 (96.8%)	
**Marital status**	Single	2554 (52.4%)	495 (67.3%)	<0.001
	Married	2185 (44.9%)	226 (30.7%)	
	Other (widowed, divorced)	131 (2.7%)	14 (1.9%)	
**Guarantor**	Displacement fund	0 (0.0%)	575 (83.9%)	<0.001
	Self-payer	968 (20.4%)	69 (10.1%)	
	Insurance	3778 (79.6%)	41 (6.0%)	
**ED Visit Characteristics**				
**Mode of arrival**	Self-transport	3005 (90.6%)	493 (81.9%)	<0.001
	Ambulance	313 (9.4%)	109 (18.1%)	
**Emergency Severity Index (ESI)**	High	407 (8.4%)	120 (15.5%)	<0.001
	Intermediate	4056 (83.3%)	597 (76.9%)	
	Low	406 (8.3%)	59 (7.6%)	
**LOS, hours**	Mean (±SD)	3.27 ± 4.67	3.85 ± 7.22	0.03
**ED Disposition**	Home	3352 (69.1%)	495 (65.0%)	<0.001
	AMA	220 (4.5%)	115 (15.1%)	
	Admit	1086 (22.4%)	49 (6.4%)	
	**General**	884 (81.4%)	30 (61.2%)	
	**Critical care**	202 (18.6%)	19 (38.8%)	
	Transfer to another facility	13 (0.3%)	11 (1.4%)	
	**General**	12 (92.3%)	9 (81.8%)	
	**Critical care**	1 (7.7%)	2 (18.2%)	
	Dead	23 (0.5%)	3 (0.4%)	
	LWBS	156 (3.2%)	89 (11.7%)	
**Labs done**	Yes	3003 (61.2%)	441 (56.8%)	0.02
**Electrocardiogram**	Yes	1721 (35.1%)	260 (33.5%)	0.40

*SD: Standard deviation, ESI: Emergency severity index, LOS: Length of stay, ED: Emergency department, AMA: against medical advice, LWBS: Left without being seen.

**Table 2:** Comparison of patient demographics and ED visit characteristics for patients admitted to critical care.

		Critical care		
		No N = 935	Yes N = 250	p-value
**Patient Demographics**				
**Age**	Mean (±SD)	56.25 ± 26.97	60.64 ± 25.85	0.009
**Age Categories**	1-17 years	113 (12.1%)	25 (10.0%)	
	18-44 years	187 (20.0%)	29 (11.6%)	
	45-64 years	181 (19.4%)	54 (21.6%)	
	65 years and older	454 (48.6%)	142 (56.8%)	
**Gender**	Male	494 (52.8%)	141 (56.6%)	0.29
	Female	441 (47.2%)	108 (43.4%)	
**Nationality**	Non-Lebanese	58 (6.2%)	22 (8.8%)	0.15
	Lebanese	877 (93.8%)	228 (91.2%)	
**Marital status**	Single	291 (31.1%)	75 (30.0%)	0.22
	Married	590 (63.1%)	167 (66.8%)	
	Other (widowed, divorced)	54 (5.8%)	8 (3.2%)	
**Guarantor**	Displacement fund	15 (1.6%)	12 (4.8%)	<0.001
	Self-payer	191 (20.5%)	81 (32.4%)	
	Insurance	727 (77.9%)	157 (62.8%)	
**ED Visit Characteristics**				
**Displaced**	Yes	39 (4.2%)	24 (9.6%)	<0.001
**Mean of arrival**	Self-transport	525 (77.7%)	121 (66.5%)	0.002
	Ambulance	151 (22.3%)	61 (33.5%)	
**Emergency Severity Index (ESI)**	High	151 (16.1%)	149 (59.8%)	<0.001
	Intermediate	781 (83.5%)	100 (40.2%)	
	Low	3 (0.3%)	0 (0.0%)	
**LOS, hours**	Mean (±SD)	5.80 ± 4.67	7.18 ± 11.11	0.03
**Labs done**	Yes	906 (96.9%)	226 (90.4%)	<0.001
**Electrocardiogram**	Yes	553 (59.1%)	197 (78.8%)	<0.001

*SD: Standard deviation, ED: Emergency department, ESI: Emergency severity index, LOS: Length of stay.

**Table 3:** ENTER multivariate logistic regression of predictors of critical care.

	Critical care (reference: no)			
	OR	95% CI		P-value
**Displaced –** Yes	2.27	1.32	3.92	**0.003**
**Age, years**	1.08	1.01	1.16	**0.03**
**LOS, hours**	1.24	1.02	1.50	**0.03**
**Gender,** Female	0.84	0.63	1.12	0.24
**Nationality,** Lebanese	0.67	0.39	1.14	0.14
**Marital status,** Married	0.97	0.66	1.41	0.86

Variables included in the model were:

Displaced (reference: no); Gender (reference: male); Age (increase by 10 years), LOS (increase by 10 hours); Nationality (reference: non-Lebanese); Marital status (reference: Single).
